# Is Early Tuberculosis Death Associated with Increased Tuberculosis Transmission?

**DOI:** 10.1371/journal.pone.0117036

**Published:** 2015-01-26

**Authors:** Anu Parhar, Zhiwei Gao, Courtney Heffernan, Rabia Ahmed, Mary Lou Egedahl, Richard Long

**Affiliations:** 1 Department of Medicine, University of Alberta, Edmonton, Alberta, Canada; 2 School of Public Health, University of Alberta, Edmonton, Alberta, Canada; 3 Department of Medicine, Memorial University of Canada, St. John’s, Newfoundland, Canada; Institut Pasteur de Lille, FRANCE

## Abstract

**Introduction:**

Tuberculosis (TB) is now a relatively uncommon disease in high income countries. As such, its diagnosis may be missed or delayed resulting in death before or shortly after the introduction of treatment. Whether early TB death is associated with increased TB transmission is unknown. To determine the transmission risk attributable to early TB death we undertook a case-control study.

**Methods:**

All adults who were: (1) diagnosed with culture-positive pulmonary TB in the Province of Alberta, Canada between 1996 and 2012, and (2) died a TB-related death before or within the first 60 days of treatment, were identified. For each of these “cases” two sets of “controls” were randomly selected from among culture-positive pulmonary TB cases that survived beyond 60 days of treatment. “Controls” were matched by age, sex, population group, +/- smear status. Secondary cases of “cases” and “controls” were identified using conventional and molecular epidemiologic tools and compared. In addition, new infections were identified and compared in contacts of “cases” that died before treatment and contacts of their smear-matched “controls”. Conditional logistic regression was used to find associations in both univariate and multivariate analysis.

**Results:**

“Cases” were as, but not more, likely than “controls” to transmit. This was so whether transmission was measured in terms of the number of “cases” and smear-unmatched or -matched “controls” that had a secondary case, the number of secondary cases that they had or the number of new infections found in contacts of “cases” that died before treatment and their smear-matched “controls”.

**Conclusion:**

In a low TB incidence/low HIV prevalence country, pulmonary TB patients that die a TB-related death before or in the initial phase of treatment and pulmonary TB patients that survive beyond the initial phase of treatment are equally likely to transmit.

## INTRODUCTION

Tuberculosis (TB) is a relatively uncommon disease in high-income countries like Canada and the United States, where crude incidence rates in 2012 were 4.8 and 3.2 per 100, 000 population, respectively [1, 2]. As a result, the diagnosis of TB can be missed or significantly delayed increasing the likelihood of morbidity and mortality in incident cases. Those most at risk of increased morbidity and mortality from TB are the elderly, in whom timely diagnosis is further challenged by atypical presentations [3–6]. Death before treatment is clearly a missed diagnosis, without assigning responsibility. Death in the initial phase of treatment is usually TB-related and therefore indicative of delayed diagnosis [7, 8]. Whether, in addition to poor individual outcomes early TB death results in poor public health outcomes as measured by an increased number of secondary cases or other transmission events, is unknown. One study involving 40 cases diagnosed after death, suggested that transmission was limited [9]. In that study, which used both conventional and molecular epidemiologic methods, only 3 cases could be linked by molecular epidemiologic methods alone to one or more secondary cases. On one hand, persons with a missed or delayed diagnosis may be expected to have more advanced disease and therefore be more infectious at presentation; on the other hand, those who die before or shortly after treatment is initiated may be expected to be older, co-morbid and possibly socially isolated, limiting the number of opportunities for transmission [5, 10]. Independent of early death, older TB patients are less likely to transmit [11, 12]. If, however, they infect older contacts, those contacts may be more likely to progress to disease on account of existent co-morbidities or intolerance to treatment of latent TB infection (LTBI). Herein we seek to address the question of whether early TB death is associated with increased transmission.

In the Province of Alberta, Canada which had a population 3,888,700 in 2012 (*Statistics Canada*), TB occurs mostly in two minority groups: (i) the foreign-born and (ii) Registered First Nations. First Nations are Native Americans; those that are registered according to the *Indian Act* of 1876 are ‘Registered’ or ‘Status’ Indians. They comprise 81% of all First Nations in Canada. Outside of these minority groups the age- and sex- adjusted incidence of TB was less than 1 per 100,000 person-years between 1998 and 2008 [13]. Within the province, the TB program is delivered out of three public health clinics, with medical expertise provided by a small group of university-based pulmonary and infectious disease physicians. Respiratory isolation of infectious cases and directly observed therapy are standard practice. HIV sero-prevalence is low and multidrug-resistant (MDR) TB is rare [14, 15]. The present study identifies all adults diagnosed in Alberta, Canada, with culture-positive pulmonary tuberculosis who died from TB either before or within the first 60 days of treatment [16, 17]. Transmission events from these “cases” and two randomly selected groups of “controls” were then compared using conventional and molecular epidemiologic methods to determine the effect of early TB death on transmission.

## METHODS

This study was approved by the Health Research Ethics Board, Panel B, at the University of Alberta. Consent was not obtained due to the retrospective nature of these data. All patient data was anonymized and de-linked prior to analysis.

All patients meeting the Canadian case definition of TB and diagnosed in the Province of Alberta are notified in a Provincial Registry [18]. Since 1996 this Registry has included information about treatment outcome and place of residence (postal code) in addition to demographic, clinical and laboratory information. Demographic information includes age, sex and population group (Canadian-born Aboriginal as defined by the *Constitution Act* of 1982, Canadian-born non-Aboriginal and foreign-born). Clinical information includes disease site (pulmonary vs. extra-pulmonary) and disease type (new active vs. relapse/retreatment). Laboratory information includes smear status (pulmonary cases), culture status, drug susceptibility test and DNA fingerprint results on initial isolates of *Mycobacterium tuberculosis*, plain chest radiographic pattern (normal or abnormal; if abnormal, cavitary or non-cavitary) and HIV status. Treatment outcome information includes whether the case is a TB death, defined as dying with TB before or during treatment of TB. TB deaths are further categorized into those in whom death was related to TB (TB was either the primary or a contributory cause of death) or those in whom death was unrelated to TB [18]. In addition to the above case-related information, the Provincial Registry also contains information about contacts of cases.

Over the 17 year period, 1996–2012, all adults (age >14 years), with culture-positive pulmonary TB who died a TB-related death before or within the first 60 days of treatment were identified in the TB Registry described above. These TB deaths are hereafter referred to as “cases” and their early death is interpreted to be indicative of missed or delayed diagnosis. To measure the effect of early TB death on TB transmission, we randomly selected a comparison group of “controls” from among those who had survived beyond 60 days of treatment. “Controls” were matched by age (+/- 5 years), sex and population group. Population group is an important variable because in Alberta Aboriginal people are more likely, and foreign-born people less likely, to transmit than others [12]. In addition, because sputum smear positivity and cavitation on chest radiograph are known to be risk factors for TB death in low TB incidence/low HIV prevalence countries such as Canada [16, 19–24], we anticipated that our “controls” might transmit less because they were less likely to have these features; sputum smear positivity and cavitation on chest radiograph also known to be independent risk factors for transmission [25–27]. To control for the effect of these features on transmission from “cases” and “controls” we randomly selected a second set of “controls”, this time matched for smear status and/or cavitation in addition to age, sex and population group. Control selection was performed using random sample selection without replacement in SAS (version 9.3).

Once “cases” and “controls” were identified, their contact lists were assembled and cross-referenced against the Registry to identify any secondary cases [18]. Secondary cases among those identified as close and casual/other contacts are described as: *type 1*, individuals diagnosed with active TB either 6 months before or 24 months after the date of diagnosis of the “case” or “control” (the transmission window), who were culture-positive and who had an isolate of *M. tuberculosis* that matched (DNA fingerprint) that of the presumed source case (see below), or *type 2*, individuals notified with active disease within the same transmission window but who were culture-negative (mainly children). The date of diagnosis of “cases” and “controls” is defined as the start date of treatment or the date of death in the event the patient died before treatment could begin. To allow for the possibility that cases who died an early TB death had incomplete contact lists, secondary cases of “cases” and, “controls” were searched for among notified cases of TB in the province who were culture-positive, had a DNA fingerprint matched isolate of *M. tuberculosis*, and were temporally (diagnosed in the same 30-month transmission window) and spatially (they lived in the same forward sortation area [FSA] as determined by the first three digits of their postal code) linked to the “case” or “control”. An FSA is a geographic unit associated with a postal facility from which mail delivery originates. In 2011 there were 153 FSAs in Alberta and 80,948 postal codes. FSAs vary in size; the largest are in rural areas [28]. These, if present, were called *type 3* secondary cases. Secondary cases that were diagnosed before the date of diagnosis of the “case” or “control” had to have had primary disease. In the event that a “case” or a “control” was themselves a secondary case of someone else, transmission events attributed to them were scrutinized for plausibility to ascertain whether their “secondary cases” were not more appropriately attributed to their own source case. “Case” and “control” groups were then compared for transmission events resulting in secondary cases. “Case” transmitters and their secondary cases are described.

The rationale behind the choice of a 30-month transmission window within which contacts could become a *type 1* or *2* secondary case was as follows. It was anticipated that most contacts that were destined to become a secondary case would do so within the period of time extending from 6 months before to 24 months after the date of diagnosis of their source “case” or “control” as the risk of disease after infection is known to be highest during this period of time. Further, it was anticipated that those contacts who were determined to be newly infected but without disease, would be offered treatment of LTBI or alternatively, followed over the subsequent 24 months. The chosen transmission window also allowed that contacts of “cases” and “controls”, each of which may have been diagnosed in a different calendar year, could be followed over a comparable period of time. Nevertheless, for purposes of establishing the validity of the 30-month transmission window, a sensitivity analysis was performed in which all contacts, regardless of the date of diagnosis of their source “case” or “control”, were followed out to December 31^st^, 2013; i.e. the temporal restriction on contact follow-up was lifted. No geospatial restriction was placed upon the contacts. It was judged that if, in sensitivity analysis, few if any additional secondary cases were found among contacts, it would be unlikely that more *type 3* secondary cases would be found outside the transmission window, as by virtue of not having been identified as contacts, they would have been beyond the reach of preventive measures and therefore yet more likely to develop disease within the transmission window.

Finally, to further establish whether early TB deaths (“cases”) were as, if not more, likely than their “controls” to transmit, the contacts of those “cases” that died before treatment and their age, sex, population group and smear-matched “controls” were grouped according to whether or not they were completely assessed and if completely assessed whether or not they were listed as a having a new positive tuberculin skin test (TST) or a TST conversion. The proportions of completely assessed contacts of “cases” and “controls” that had a new positive TST or a TST conversion were also compared.

### DNA fingerprinting methodology

Isolates of *M. tuberculosis* from all culture-positive cases of TB diagnosed in the Province of Alberta are routinely fingerprinted using standardized restriction fragment-length polymorphism (RFLP), supplemented in those isolates with five or fewer copies of the insertion sequence *6110*, by spoligotyping [29, 30]. Images were digitized using the imager video camera system (Appligene, Illkirch, France). Digitized gel images were analyzed using Gelcompar II software (Applied Maths, Kortrijk, Belgium). The analysis is performed on coded specimens in blinded fashion. All isolates matched as identical by computer were manually confirmed by visually comparing the original autoradiographs. Between 1996 and 2012, 99.3% of all initial isolates of *M. tuberculosis* (n = 1978) were DNA fingerprinted. From July to December 1995 and January to December 2013, months that were included in the transmission windows of some “cases” and “controls”, 100% of isolates were DNA fingerprinted.

### Statistical analysis

Conditional logistic regression is used to examine the association between outcome and transmission with risk factors in univariate analysis and multivariate analysis. Odds ratios and 95% confident intervals were calculated. All statistical analyses were carried out by SAS.

## RESULTS

**Figure 1:**
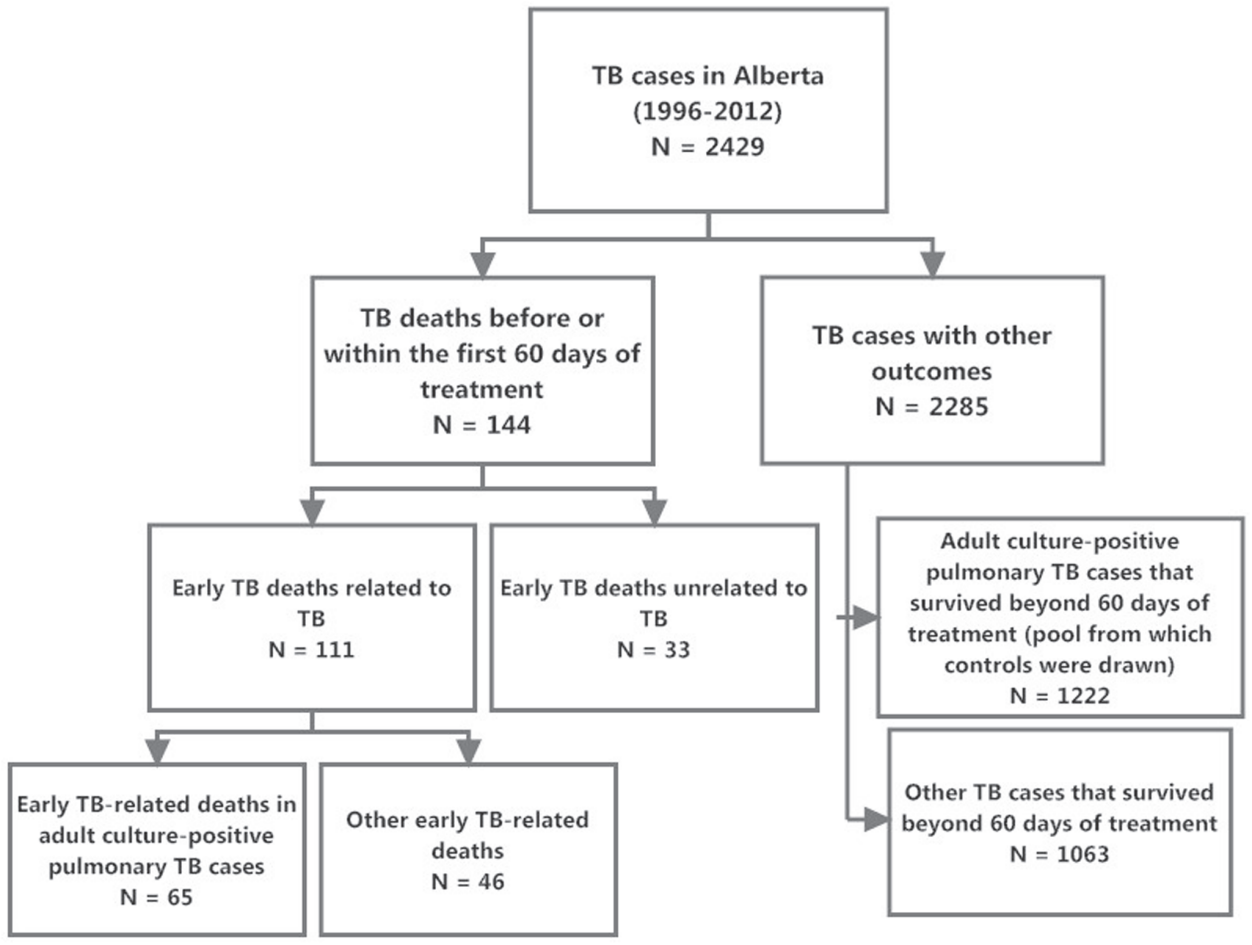
TB Cases in Alberta from 1996–2012: Cases with Early Death and Other Outcomes.

In Alberta, between 1996 and 2012, 144 of the total of 2429 individuals diagnosed with TB (5.9%), died before or within the first 60 days of treatment (see Fig. 1). Of the 144 early TB deaths, 111 (77.1%) were TB-related and of the 111 TB-related deaths, 65 (58.6%) had culture-positive pulmonary TB and were over 14 years of age (see Table 1). Among these early adult culture-positive pulmonary TB deaths 15 (23.1%) died before treatment and 50 (76.9%) died within the first 60 days of treatment. These individuals comprise the “cases” in this case-control study. Most were older (74% were >64 years of age), male (60%) and either Canadian-born Aboriginal or foreign-born (89%), see Table 2.

**Table 1:** Adult culture-positive pulmonary TB cases that died either before or within the first 60 days of treatment, according to time of death and cause of death (“cases” in the case-control analysis).

**Cases**	**Cause of death of pulmonary TB cases**		**Total n (%)**
	**TB was the primary cause of death n (%)**	**TB was a contributory cause of death n (%)**	
Death before treatment	2 (13.3)	13 (26.0)	15 (23.1)
Death in the first 30 days of treatment	11 (73.3)	28 (56.0)	39 (60.0)
Death after 30 and before 60 days of treatment	2 (13.3)	9 (18.0)	11 (16.9)
Total	15 (100.0)	50 (100.0)	65 (100.0)

**Table 2:** Demographic and clinical characteristics of pulmonary tuberculosis patients who died before or within 60 days of treatment (cases) or survived beyond 60 days of treatment (controls).

**Characteristic**	**“Cases” n (%)**	“Controls”*	
		**Unmatched for smear n (%)**	**Matched for smear n (%)**
**Number Assessed**	65	130	130
**Age at diagnosis (years)**			
15–64	17 (26)	33 (25)	32 (25)
>64	48 (74)	97 (75)	98 (75)
**Sex**			
Male	39 (60)	77 (59)	79 (61)
Female	26 (40)	53 (41)	51 (39)
**Population Group**			
Canadian-Born Aboriginal^†^	16 (25)	32 (25)	32 (25)
Canadian-Born Non-Aboriginal	7 (11)	14 (11)	14 (11)
Foreign-born	42 (65)	84 (65)	84 (65)
**Disease Type**			
New Active	61 (94)	113 (87)	105 (81)^‡^
Relapse/Retreatment	4 (6)	17 (13)	25 (19)
**Smear Positive**			
Yes	40 (62)	64 (49)	80 (62)
No	25 (38)	66 (51)	50 (38)
**Cavitary Disease**			
Yes	11 (17)	28 (22)	27 (21)
No	54 (83)	102 (78)	103 (79)
**Drug-resistant^§^**			
Yes	0 (0)	0 (0)	1 (1)
No	65 (100)	130 (0)	129 (99)
**HIV positive**			
Yes	3 (5)	3 (2)	3 (2)
No	19 (29)	72 (55) ‡	59(45)
Unknown	43 (66)	55 (42)	68 (53)

*One set of controls was matched by age (± 5 years), sex and population group. Another set of controls was matched by age, sex, population groups and smear status. Among the controls matched by age (± 5 years), sex, population group and smear status there were 8 that could only by matched by age ± 10 years

^†^Canadian-born Aboriginal includes individuals identified as First Nations, Métis, or Inuit according to the *Constitution Act* of 1982

^‡^p < 0.05

^§^Only multidrug-resistant cases (defined as resistance to isoniazid and rifampin with or without resistance to other drugs), were included.

Compared to “controls”, “Cases” were 1.3 times (p = 0.11) more likely to have smear-positive disease but no more likely to have cavitary disease (Table 2). Accordingly, to control for any effect of smear status on transmission a second set of “controls” was randomly selected, this time being matched by smear status in addition to age, sex and population group (Table 2). “Cases” were less likely than smear-matched “controls” to have relapse/retreatment disease and “cases” were less likely than both sets of “controls” to be HIV tested (33.8% of “cases” vs 57.7% of unmatched “controls” vs 47.7% of matched “controls”, respectively). Among those who were HIV tested there was a trend toward “cases” being more likely than “controls” to be HIV positive (13.6% of “cases” vs 4.0% of unmatched “controls” vs 4.8% of matched “controls”), but the numbers were small. “Cases” were no more likely than “controls” to have MDR-TB. The mean, median, and interquartile range (IQR) diagnosis year for “cases” was 2003.9, 2004 and 7, respectively; for unmatched “controls” 2004.2, 2005 and 10, respectively; and for matched “controls” 2003.3, 2003 and 10, respectively. Among “controls” that were unmatched and matched for smear status 9 (6.9%) and 8 (6.2%), respectively, died after the first 60 days of treatment with TB as a primary or contributory cause of death. To strengthen any conclusions that might be drawn about transmission from “cases” and “controls”, the analysis was performed using both sets of “controls”.

Before running the analysis, contact lists of “cases” and both sets of “controls” were assembled and organized by age, sex and “case”/“control” smear status (see Table 3). With just two exceptions there were no differences in the mean and median number of close contacts of “cases” and “controls”. The two exceptions were the number of close contacts of “controls” over 4 years of age and the number of close male contacts of “controls” matched for smear, where the number of contacts of “controls” was less than the number of contacts of “cases”. The number of casual/other and total contacts of “cases” was greater among “cases” that were >4 years of age, “cases” that were both male and female and “cases” that were smear-positive, when compared to their corresponding “controls”. Among “cases” that died before treatment (n = 15) and “cases” that died within the first 60 days of treatment (n = 50), there were no significant differences in the mean/median/IQR number of close (15.4/11/10 vs 15.1/10/11, p = 0.93) or casual/other contacts (16.5/7/28 vs 31.0/13/37, p = 0.30).

**Table 3:** Identified contacts of “cases” and “controls” by age, sex and smear status of source case or control.

**Characteristics**	**“Case” contacts**	**“Control” Contacts**	
		**Unmatched for smear**	**Matched for Smear**
	**Mean/Median(IQR)**	**Mean/Median(IQR)**	**Mean/Median(IQR)**
**Age**			
<5 years			
Close	1.4/0(1)	0.8/0(1)	1/0(1)
Casual/Other	1.3/0(2)	0.7/0(1)*	2/0(1)
Total	2.6/1(3)	1.5/0(2)	2/1(2)
≥5 years			
Close	14/9(13)	9/5(8)*	9/5(7)*
Casual/Other	26/11(28)	23/1(13)*	13/2(12)*
Total	39/26(35)	33/8(22)*	22/9(17)*
**Sex**			
Male			
Close	6/4(6)	5/3(5)	4/3(4)*
Casual/Other	10/3(8)	8/0(4)*	5/1(5)*
Total	16/8(11)	12/4(8)*	9/5(9)*
Female			
Close	9/5(8)	5/3(4)	6/3(4)
Casual/Other	17/7(23)	16/0(7)*	9/1(7)*
Total	25/16(26)	21/4(11)*	14/5(10)*
**Smear status of source**			
Smear-positive			
Close	19/12.5(18)	14/8(14)	13/6(11)
Casual/Other	39/16.5(49)	46/10(29)*	22/7.5(22)*
Total	58/35.5(54)	60/21(56)*	35/16(45)*
Smear-negative			
Close	10/7(8)	6/4(6)	6/4(6)
Casual/Other	10/2(11)	3/0(1)	5/0(3)
Total	19/11(19)	10/5(6)	11/6(8)

* p < 0.05: conditional logistic regression with logarithm transformation of number of contacts. We imputed 0.01 as the number of contacts for patients who have no contact.

Among the contacts of both “cases” and “controls” there were not many *type 1, 2* secondary cases. Out of the total of 65 “cases” only 4 (6.2%), had *type 1* or *2* secondary cases. Out of the total of 130 unmatched-for-smear and the total of 130 matched-for-smear “controls” only 5 (3.8%) and 4 (3.1%), respectively, had *type 1* or *2* secondary cases (see Table 4). As well, very few “cases” and “controls” had *type 3* secondary cases; only 1 “case” and 3 unmatched-for-smear and 2 matched-for-smear “controls” had *type 3* secondary cases. Neither the number of “case” and “control” transmitters nor the number of *type 1*, *2* or *3* secondary cases they had (8, 9 and 6 secondary cases from “cases” and unmatched and matched “controls”, respectively), was significantly different. “Case” transmitters and their *type 1, 2*, and *3* secondary cases are described in detail in Table 5. All were ≥64 years of age, smear-positive and co-morbid. All died within the first 30 days of treatment. In none did it appear chemotherapy was a contributor to death.

**Table 4:** Secondary cases resulting from pulmonary TB patients who died before or within the first 60 days of treatment (“cases”) and those who survived beyond 60 days of treatment (“controls”).

Secondary Cases*	**“Cases”**		**“Controls”**					
			**Unmatched for Smear**			**Matched for Smear**		
	**Without secondary Cases**	**With Secondary Cases**	**Without Secondary Cases**	**With Secondary Cases**	**OR (95%CI)**	**Without Secondary Cases**	**With Secondary Cases**	**OR (95%CI)**
	**n = 60**	**n = 5**	**n = 122**	**n = 8**		**n = 124**	**n = 6**	
*Type I*		4		3	1.8 (0.8,4.3)		2	2.0 (0.9,4.7)
*Type II*		2		3	1.4 (0.5,3.8)		2	1.4 (0.5,3.8)
*Type III*		2		3	0.8 (0.2, 2.7)		2	1.0 (0.3,3.3)
Total		8		9	1.1 (0.6,2.1)		6	1.3 (0.7,2.5)

* See text for definition of *type 1, 2* and *3* secondary cases

**Table 5:** Characteristics of early TB deaths that were “case transmitters” and characteristics of their secondary cases.

**“Case” Transmitters**								**Secondary Cases**						
**“Case”**	**Age**	**Sex**	**Pop. Group**	**Smear Status**	**Days Tx**	**Place of Dx**	**Co-morbidities**	Type of 2°Case*	**Age**	**Sex**	**Pop. Group**	**Disease Site**	**Time to Dx†**	**Co-morbidities**
A	64	F	CBA	+	2	hosp	Adult Respiratory Distress Syndrome	1	22	F	CBA	primary pulmonary	12	depression
							Septic shock Acute renal failure coagulopathy	2	2	M	CBA	primary pulmonary	5	
B	86	F	CBA	+	8	hosp	Acute myocardial infarct; congestive heart failure; CVA;diabetes	1	1	M	CBA	primary pulmonary	6	Asthma; iron deficiency anemia
							Cerebro-vascular accident; anemia COPD	2	10	M	CBA	primary pulmonary	17	Beckwith-Wiedeman Syndrome; gastro-eosophageal reflux
C	88	F	FB	+	19	hosp	COPD	1	62	F	FB	pulmonary	43	
D	90	M	FB	+	8	hosp	Diabetes;Chronic renal insufficiency; Malnutrition; Atrial fibrillation	1	16	M	CBO	primary pulmonary	32	
E	85	M	CBA	+	1	hosp	Atrial fibrillation and congestive heart failure (probable)	3	72	F	CBA	pulmonary	133	Gastric carcinoma; diabetes; coronary disease; CVA
								3	68	F	CBA	bone and joint	552	Dialysis-dependent renal failure; diabetes

Abbreviations: M male; F female; CBA Canadian-born Aboriginal; CBO Canadian-born “Other”; FB Foreign-born; Tx treatment; Dx diagnosis; Hosp hospital; COPD Chronic Obstructive Pulmonary Disease; CVA cerebro-vascular accident

*See text for definitions of type of secondary case.

†Time to diagnosis is the number of days between the diagnosis of the “case transmitter” and the diagnosis of the secondary case.

In sensitivity analysis, only one additional secondary case was found among the contacts of “cases” (a *type 1* secondary case that was diagnosed 5.8 years after a “case” that had previously been identified as a transmitter) and only one additional secondary case was found among the contacts of “controls” (a *type 2* secondary case that was diagnosed 3 years after a matched-for-smear “control” that had not previously been identified as a transmitter). These results further validated our methodology and lent support to our conclusions.

Finally, evidence of infection (new positive TST or TST conversion) rather than disease (secondary case) was analyzed among the close and casual/other contacts of “cases” that died before treatment and their smear-matched “controls”. The mean and median age of these “cases” was 76.6 and 77 years, respectively; 40% were smear-positive (see Table 6). Among completely assessed close and casual/other contacts, the proportion with a new positive TST or TST conversion was not significantly different in “cases” or “controls”.

**Table 6:** Tuberculin skin test results in contacts of “cases” that died before treatment (n = 15) and their matched (for age, sex, population group and smear status) “controls” (n = 30).

**Results**	**Contacts of “Cases”**	**Contacts of “Controls”**	**P-value**
**No. Contacts**			
Close	225	301	
Casual/Other	241	408	
Total	466	709	
**No. Contacts Completely Assessed (% of total)**			
Close	81.4	90.5	0.18
Casual/Other	74.2	82.3	0.31
Total	76.3	86.2	0.10
No. new positive TSTs among contacts that were completely assessed (% of completely assessed)*			
Close	19.2	16.7	0.65
Casual/Other	5.5	17.7	0.07
Total	12.2	14.9	0.45
No. TST converters among contacts that were completely assessed (% of completely assessed)*^†^			
Close	1.8	2.2	0.79
Casual/Other	1.6	1.6	0.96
Total	2.7	2.6	0.96

Abbreviations: TST tuberculin skin test

* New positive TSTs and TST converters were defined according to the Canadian TB Standards, 7^th^ Edition

† TST converters include those contacts diagnosed with prevalent active TB

## DISCUSSION

In this study we began with the assumption that early TB death is a proxy for missed or delayed diagnosis of TB. An early TB death was defined as an adult culture-positive pulmonary TB “case” that died before or within the first 60 days of treatment (initial phase), with TB understood to be either the primary or a contributory cause of death. We found that “cases” were as likely, but not more likely, than age (+/- 5 years), sex, population group and smear-status unmatched or matched “controls”, to transmit. This was so whether transmission was measured in terms of the number of “cases” and “controls” that had a secondary case, the number of secondary cases that they had, or the number of new infections (new positive TSTs or TST conversions) that were found in close and casual/other contacts of those who died before any treatment. Although there was a trend towards early TB deaths being more likely to transmit to their contacts (4/65 “cases” [6.2%] vs 5/130 unmatched [3.8%] and 4/130 matched [3.1%] “controls”, respectively, had one or more *type 1* or *2* secondary cases), this finding was not statistically significant. Nor was it supported by our *type 3* secondary case (DNA fingerprint-matched cases that could be temporally and spatially linked to an early TB death, the inference being that they were unidentified contacts) analysis, where only 1 “case” and 3 smear-unmatched and 2 smear-matched “controls” had one or more secondary cases. That neither “cases” nor “controls” had many secondary cases, *types 1, 2* or *3*, is perhaps best explained by the age and population group of both “cases” and “controls”; most were >64 years of age (~75%) and foreign-born (65%). Below we ask whether it is indeed reasonable to assume that early TB deaths had a missed or delayed diagnosis and if so why they do not appear to transmit any more than their “controls”.

Given the preventable and curable nature of TB we believe it makes sense to interpret the early death of our “cases” as a diagnostic delay. Our “cases” were: (i) significantly less likely than their smear-matched “controls” to have relapse or retreatment disease, disease types that warn of TB, (ii) 1.3 times more likely than smear-unmatched “controls” to be smear-positive suggesting more advanced disease, and (iii) in contact with more persons than their “controls” suggesting a longer period of activity prior to diagnosis, though it is safe to assume that many TB deaths were hospitalized prior to diagnosis where it may be predicted large numbers of susceptible (and possibly vulnerable) contacts would be exposed over relatively short periods of time [5, 31–33]. In low TB incidence/low HIV prevalence countries the risk of a TB patients’ dying is increased if they have been symptomatic for >4 weeks [23, 24, 34]. But can we say that “cases” were symptomatic longer than their “controls”? Our transmission results suggest otherwise.

The apparent contradiction between a “case” having a missed or delayed diagnosis and a “case” being no more likely than a “control” to transmit is best explained by differential co-morbidity rates in the two groups. If, as a group, our TB deaths were more likely than their “controls” to have co-morbidities, they would be less able to compensate for any delay, however long, or complication. A similar delay, with similar transmission, might result in early TB death in a co-morbid “case” but not in a non-co-morbid “control”. Although information on co-morbidities was not systematically collected in our study, differential co-morbidity rates are highly likely given that non-infective co-morbidities such as malignancy, renal disease, malnutrition, etc are risk factors for TB death in low TB incidence/low HIV prevalence countries, and given that the primary cause of death in 50 (76.9%) of our early TB deaths was a medical condition other than TB [10, 19, 20, 23, 35–38]. Among co-morbidities those that are risk factors for reactivation of LTBI serve the pathogen but not the host; if those same co-morbidities are risk factors for death (especially in the elderly), they serve neither the pathogen nor the host.^18^ Not excluded is the possibility that co-morbid “cases” even if symptomatic for longer than “controls”, may be less able to generate a cough aerosol because of weakness, reduced cough reflex, dehydration etc [39].

Many years ago Stead et al demonstrated very convincingly that elderly debilitated pulmonary TB patients have the ability to transmit [40, 41]. Their observations were confirmed in our own study where five early TB deaths, all ≥ 64 years of age, were shown to have secondary cases and where fifteen early TB deaths (those that died before any treatment; mean and median age 76.6 and 77 years, respectively) were shown to be just as likely to infect contacts as were their “controls”. That early TB deaths transmitted as much as their “controls” substantiates all earlier claims registered in the literature about transmission from early TB deaths, and the imperative to make a timely diagnosis. It suggests that the screening of contacts of early TB deaths should proceed apace as for any other smear and/or culture-positive pulmonary TB case.

Obvious limitations of our study include the relatively small number of early adult culture-positive pulmonary TB-related deaths (n = 65), especially the number that died without any treatment (n = 15) and the assumptions that we made in lieu of actual data on the co-morbidity rate and living arrangements of “cases” and “controls” at the time of diagnosis. With respect to the former, information on co-morbidities was not systematically collected during the years of the study. With respect to the latter, we did not know what proportion of “cases” versus “controls”, were institutionalized at the time of diagnosis. In particular, were they hospitalized and if so for how long? In another Canadian study, Xie et al reported that, among cases diagnosed after death, 75% were hospitalized prior to death, several for periods of two weeks or more [33]. Institutionalization prior to death might explain why those of our early TB deaths that died without any treatment had just as many contacts as their “controls”. In San Francisco, DeRiemer et al found that those diagnosed after death had significantly fewer contacts than those diagnosed alive [9]. However, the design and context of their study and our own may not be comparable.

In summary, in a low TB incidence/low HIV prevalence country, pulmonary TB patients that die before or in the initial phase of treatment and pulmonary TB patients that survive beyond the initial phase of treatment, are equally likely to transmit. Differential co-morbidity rates might explain why early TB deaths do not transmit more than “control” patients who are not early TB deaths. Early TB death is doubly bad; it is a most unfortunate outcome for the individual and it is a contributor to the persistence of TB in low incidence countries.

## References

[1] The Public Health Agency of Canada (2013) Tuberculosis in Canada 2012-Pre-release. Ottawa (Canada): Minister of Public Works and Government Services Canada.

[2] CDC (2013) Reported Tuberculosis in the United States, 2012. Atlanta, GA: U.S. Department of Health and Human Services CDC, 10.

[3] Anees Khan MA, Kovnat DM, Bachus B, Whitcomb ME, Brody JS, et al (1977) Clinical and radiographic spectrum of pulmonary tuberculosis in the adult. Am J Med 62: 31–38. 10.1016/0002-9343(77)90346-1 835590

[4] Miller WT, MacGregor RR (1978) Tuberculosis frequency of unusual radiographic findings. AJR 130: 867–875. 10.2214/ajr.130.5.867 417585

[5] Naalsund A, Heldal AL, Johansen B, Kongerud J, Boe J (1994) Deaths from pulmonary tuberculosis in a low-incidence country. J Intern Med 236: 137–142. 10.1111/j.1365-2796.1994.tb01275.x 8046312

[6] Pérez-Guzman C, Vargas MH, Torres-Cruz A, Villarreal-Velarde H (1999) Does aging modify pulmonary tuberculosis? A meta-analytical review. CHEST 116: 961–967. 10.1378/chest.116.4.961 10531160

[7] Murray J, Sonnenberg P, Nelson G, Bester A, Shearer S, et al (2007) Cause of death and presence of respiratory disease at autopsy in an HIV-1 sero-conversion cohort of South African Gold Miners. AIDS 21: S97–S104. 10.1097/01.aids.0000299416.61808.24 18032945

[8] Mukadi YD, Maher D, Harries A (2001) Tuberculosis case fatality rates in high HIV prevalence populations in Sub-Saharan Africa. AIDS 15: 143–152. 10.1097/00002030-200101260-00002 11216921

[9] DeRiemer K, Rudoy I, Schecter F, Hopewell PC, Daley C (1999) The epidemiology of tuberculosis diagnosed after death in San Francisco, 1986–1995. Int J Tuberc Lung Dis 3: 488–493. 10383061

[10] Walpola H, Siskind V, Patel M, Konstantinos A, Dehry P (2003) Tuberculosis-related deaths in Queensland, Australia, 1989–1998: characteristics and risk factors. Int J Tuberc Lung Dis 7: 742–750. 12921150

[11] Borgdorff MW, Nagelkerke NJD, de Haas Pew, van Soolingen D (2001) Transmission of *Mycobacterium tuberculosis* depending on the age and sex of source cases. Am J Epidemiol 154: 934–943. 10.1093/aje/154.10.934 11700248

[12] Kunimoto D, Sutherland K, Manfreda J, Wooldrage K, Fanning A, et al (2004) Transmission characteristics of tuberculosis in the foreign-born and Canadian-born population of Alberta, Canada. Int J Tuberc Lung Dis 8: 1213–1220. 15527153

[13] Jensen M, Lau A, Langlois-Klassen D, Boffa J, Manfreda J, et al (2012) A population-based study of TB epidemiology and innovative service delivery in Canada. Int J Tuberc Lung Dis 16: 43–49. 10.5588/ijtld.11.0374 22236844

[14] Long R, Niruban J, Heffernan C, Cooper R, Fisher D, et al (2014) “A 10-year population-based study of ‘Opt-out’ HIV testing of tuberculosis patients in Alberta, Canada: National implications”. PLoS One 9: e98993 10.1371/journal.pone.0098993 24911262 PMC4049754

[15] Long R, Langlois-Klassen D (2013) Increase in multidrug-resistant tuberculosis (MDR-TB) in Alberta among foreign-born persons: implications for tuberculosis management. Can J Public Health 104: e22–e27. 23618116 10.1007/BF03405649 PMC6973612

[16] Waitt CJ, Squire SB (2011) A systematic review of risk factors for death in adults during and after tuberculosis treatment. Int J Tuberc Lung Dis 15: 871–885. 10.5588/ijtld.10.0352 21496360

[17] Yen Y-F, Yen M-Y, Shih H-C, Hu B-S, Ho B-L, et al (2013) Prognostic factors associated with mortality before and during anti-tuberculosis treatment. Int J Tuberc Lung Dis 17: 1310–16. 10.5588/ijtld.12.0888 24025383

[18] The Canadian Lung Association and the Public Health Agency of Canada. Canadian Tuberculosis Standards. 6thEd. Ottawa, ON, Canada: CLA and PHAC, 2007 Available: http://publications.gc.ca/collections/collection_2011/aspc-phac/HP40-18-2007-eng.pdfAccessed 2014 October 1.

[19] Shen X, DeRiemer K, Yuan Z, Shen M, Xia Z, et al (2009) Deaths among tuberculosis cases in Shanghai, China: who is at risk? BMC Infect Dis 9: 95 10.1186/1471-2334-9-95 19531267 PMC2702371

[20] Dewan PK, Arguin PM, Kiryanova H, Kondroshova NV, Khorosheva TM, et al (2004) Risk factors for death during tuberculosis treatment in Orel, Russia. Int J Tuberc Lung Dis 8: 598–602. 15137537

[21] Mathew TA, Ovsyanikova TN, Shin SS, Gelmanova I, Balbuena DA, et al (2006) Causes of death during tuberculosis treatment in Tomsk Oblast, Russia. Int J Tuberc Lung Dis 10: 857–863. 16898369

[22] Cullinan P, Meredith SK (1991) Deaths in adults with notified pulmonary tuberculosis 1983–5. Thorax 46: 347–350. 10.1136/thx.46.5.347 2068691 PMC463133

[23] Fielder JF, Chaulk CP, Dalvi M, Gachuhi R, Comstock GW, et al (2002) A high tuberculosis case-fatality rate in a setting of effective tuberculosis control: implications for acceptable treatment success rates. Int J Tuberc Lung Dis 6: 1114–1117. 12546121

[24] Kourbatova EV, Borodulin BE, Borodulina EA, del Rio C, Blumberg HM, et al (2006) Risk factors for mortality among adult patients with newly diagnosed tuberculosis in Samara, Russia. Int J Tuberc Lung Dis 10: 1224–1230. 17131780

[25] Catanzaro A (1982) Nosocomial Tuberculosis. Am Rev Respir Dis 125: 559–562. 7081816 10.1164/arrd.1982.125.5.559

[26] Bailey WC, Gerald LB, Kimmerling ME, Redden D, Brook N, et al (2002) Predictive model to identify positive tuberculosis skin test results during contact investigations. JAMA 287: 996–1002. 10.1001/jama.287.8.996 11866647

[27] Madhi F, Fuhrman C, Monnet I, Atassi K, Poirier C, et al (2002) Transmission of Tuberculosis from adults to children in a Paris suburb. Pediatr Pulmonol 34: 159–163. 10.1002/ppul.10153 12203843

[28] Government of Canada, Statistics Canada, “Postal Code”. Available: http://www12.statcan.gc.ca/census-recensement/2011/ref/dict/geo035-eng.cfm. Accessed 2014 October 1.

[29] Van Embden JDA, Cave MD, Crawford JT, Dale JW, Eisenach KD, et al (1993) Strain identification of *Mycobacterium tuberculosis* by DNA fingerprinting recommendations for a standardized methodology. J Clin Microbiol 31: 406–409. 8381814 10.1128/jcm.31.2.406-409.1993 PMC262774

[30] Dale JW, Brittain D, Cataldi AA, Cousins D, Crawford JT, et al (2001) Spacer oligonucleotide typing of bacteria of the Mycobacterium tuberculosis complex recommendations for standardized nomenclature. Int J Tuberc Lung Dis 5: 216–219. 11326819

[31] Enarson DA, Grzybowski S, Dorken E (1978) Failure of diagnosis as a factor in tuberculosis mortality. CMAJ 118: 1520–1522. 657048 PMC1818119

[32] Katz I, Rosenthal T, Michaeli D (1985) Undiagnosed Tuberculosis in Hospitalized Patients. CHEST 87: 770–776. 10.1378/chest.87.6.770 3996065

[33] Xie HJ, Enarson DA, Chao CW, Allen EA, Gryzbowski S (1992) Deaths in tuberculosis patients in British Columbia, 1980–1984. Tuber Lung Dis 73: 77–82. 10.1016/0962-8479(92)90059-S 1643301

[34] Zahar JR, Azoulay E, Klement E, De Lassence A, Lucet JC, et al (2001) Delayed treatment contributes to mortality in ICU patients with severe active pulmonary tuberculosis and acute respiratory failure. Intensive Care Med 27: 513–520. 10.1007/s001340000849 11355119 PMC7095425

[35] Rao VK, Iademarco EP, Fraser VJ, Kollef MH (1998) The impact of co-morbidity on mortality following in-hospital diagnosis of tuberculosis. CHEST 114: 1244–1252. 10.1378/chest.114.5.1244 9823996

[36] Oursler KK, Moore RD, Bishai WR, Harrington SM, Pope DS, et al (2002) Survival of patients with pulmonary tuberculosis: clinical and molecular epidemiologic factors. Clin Infect Dis 34: 752–759. 10.1086/338784 11850859

[37] Erbes R, Oettel K, Raffenberg M, Mauch H, Schmidt-Ionas M, et al (2006) Characteristics and outcome of patients with active pulmonary tuberculosis requiring intensive care. Eur Respir J 27: 1223–1228. 10.1183/09031936.06.00088105 16481385

[38] Lubart E, Lidgi M, Leibovitz A, Rabinovitz C, Segal R (2007) Mortality of patients hospitalized for active tuberculosis in Israel. Isr Med Assoc J 9: 870–873. 18210928

[39] Jones-López EC, Namugga O, Mumbowa F, Ssebidandi M, Mbabazi O, et al (2013) Cough aerosols of *Mycobacterium tuberculosis* predict new infection. Am J Respir Crit Care Med 187: 1007–1015. 10.1164/rccm.201208-1422OC 23306539 PMC3707366

[40] Stead W (1981) Tuberculosis among elderly persons: an outbreak in a nursing home. Ann Intern Med 94: 606–610. 10.7326/0003-4819-94-5-606 7235393

[41] Stead WW, Lofgren JP, Warren E, Thomas C (1985) Tuberculosis as an endemic and nosocomial infection among the elderly in nursing homes. N Engl J Med 312: 1483–1487. 10.1056/NEJM198506063122304 3990748

